# Mechanisms of Pathogen and Pesticide Resistance in Honey Bees

**DOI:** 10.1152/physiol.00033.2023

**Published:** 2024-02-27

**Authors:** Leonard J. Foster, Nadejda Tsvetkov, Alison McAfee

**Affiliations:** Department of Biochemistry and Molecular Biology and Michael Smith LaboratoriesUniversity of British Columbia, Vancouver, British Columbia, Canada

**Keywords:** Apis mellifera, honey bee, Paenibacillus larvae, Varroa destructor

## Abstract

Bees are the most important insect pollinators of the crops humans grow, and *Apis mellifera*, the Western honey bee, is the most commonly managed species for this purpose. In addition to providing agricultural services, the complex biology of honey bees has been the subject of scientific study since the 18th century, and the intricate behaviors of honey bees and ants, fellow hymenopterans, inspired much sociobiological inquest. Unfortunately, honey bees are constantly exposed to parasites, pathogens, and xenobiotics, all of which pose threats to their health. Despite our curiosity about and dependence on honey bees, defining the molecular mechanisms underlying their interactions with biotic and abiotic stressors has been challenging. The very aspects of their physiology and behavior that make them so important to agriculture also make them challenging to study, relative to canonical model organisms. However, because we rely on *A. mellifera* so much for pollination, we must continue our efforts to understand what ails them. Here, we review major advancements in our knowledge of honey bee physiology, focusing on immunity and detoxification, and highlight some challenges that remain.

## Introduction

Humans have depended on honey bees^1^ for millennia, first for their honey and then, as we grew more agrarian, for pollinating crops. While there are >20,000 species of bees described (1), only a few dozen of these make surplus honey that humans can eat, and even fewer still have been domesticated as managed pollinators. Two species, *Apis mellifera* and *A. cerana*, are by far the most significant for commercial honey production and managed pollination, with *A. mellifera* being the dominant species across most of the world. Both species have colonies made up of tens of thousands of workers (sterile females), hundreds to thousands of seasonally produced drones (males), and a single queen (reproductive female), each with strong physiological differences; however, the specifics of these differences are often poorly defined, with a marked bias toward studying the worker caste.

The long, mutualistic relationship with humans has meant that honey bees have also been the subject of extensive scientific study. Through much of the twentieth century, research focused on the fascinating suite of behaviors that make honey bees one of the few groups of eusocial animals and such successful pollinators. In 1973, Karl von Frisch (2) earned one of the few Nobel Prizes in Physiology or Medicine awarded for a topic completely unrelated to human health: deciphering the honey bee waggle dance language. Our curiosity about honey bees has extended into the genomics era, starting with the publication of the initial draft genome of *A. mellifera* in 2006 (3), which made it the third insect to have its genome sequenced, after *Anopheles gambiae* (a mosquito species complex that is the primary vector of malaria in Africa) and *Drosophila melanogaster* (the dominant model system for genetic studies) (4, 5).

Around the same time, the health of honey bees rocketed into the public consciousness when beekeepers started reporting high rates of colony losses (6). We did not understand the causes of these losses initially, leading to the term “colony collapse disorder” as well as abounding speculation about a new, devastating disease of bees (7). With more time and research, we came to understand that virtually all the colony losses could be explained by causes that were already known, albeit exacerbated by several factors (8, 9). However, annual colony losses have generally remained high, and the underlying causes typically fall into a few categories: pathogens and parasites (particularly those developing resistance to treatments), xenobiotics, weather, and beekeeping mistakes. The latter two are more incidental effects that we will not discuss further here. Instead, we will discuss how honey bees are affected by, respond to, and defend against pathogens, parasites, and xenobiotics, focusing on the physiology of *A. mellifera*, the most extensively studied bee species.

## Honey Bee Parasites and Pathogens

### Parasitic Mites

By far the most significant cause of honey bee deaths over the past two decades has been the ectoparasitic mite *Varroa destructor*. However, *Varroa*^2^ is not a natural pest of *A. mellifera*: at least a few times in history, *Varroa* jumped to *A. mellifera* from *A. cerana* when beekeepers brought the two species into close proximity (10). With only a few decades to adapt, *A. mellifera* has not yet evolved sufficient resistance to the mite, although such traits do exist in the population and can be enriched through selective breeding (11). *Varroa* has rather specific host tropism: it is only known to infest *A. mellifera* and *A. cerana*, with its life cycle closely aligned with these two closely related bee species. Briefly, a mated female (foundress) mite enters a bee brood cell before the larva has spun its cocoon, then mature mite progeny emerge from the cocoon with the host bee. This next generation of mites go on to parasitize other larvae or adult bees, upon whose hemolymph and fat body they feed (12). This feeding on host tissues damages honey bees directly through wounding and sapping nutrients, but it can also result in virus transmission. Much like how mosquitoes can vector viruses like Zika, dengue, or yellow fever, *Varroa* mites transmit viruses between individual honey bees (13–16) and these can be at least as damaging to the honey bees as the parasitism. However, *Varroa* is not merely a mechanical vector; it is also a biological vector, with at least some viruses actively replicating within the mite (17). Interestingly, the viruses may not be entirely benign to their vector, as evidenced by deformed wing virus B seeming to reduce the lifespan of *Varroa*, too (18).

### Honey Bee Viruses

Although dozens of viruses infect honey bees (reviewed in Refs. 9, 19, 20), with more still being described (21), the most common viral pathogens of *A. mellifera* belong to the Dicistroviridae and Iflaviridae families. These include deformed wing virus (A and B subtypes), black queen cell virus, acute bee paralysis virus, Israeli acute paralysis virus, chronic bee paralysis virus, and sacbrood virus, among others. The gross anatomical effects of these are varied, from eliciting no overt effect to causing developmental abnormalities or severe paralysis, but the molecular pathology of all of them remains poorly understood. Israeli acute paralysis virus, which is developing into a model for dicistroviruses, is perhaps the best understood at the molecular level (22, 23). While we refer to these as “honey bee viruses,” this is simply indicative of the host-of-first-detection, and many actually have broad host ranges across other arthropods (24) and possibly even plants (25). There are not yet any approved treatments for honey bee viruses.

*Dicistroviridae* are positive-strand RNA viruses that encode an RNA-dependent RNA polymerase for replicating their genome. These viruses hijack host protein synthesis machinery and effectively shut down the synthesis of host proteins in favor of viral proteins. Although data supporting this feature were largely derived from other insect dicistroviruses, it is likely a conserved feature in honey bee viruses as well (26). As the family name suggests, viruses in this group have two open reading frames, both of which encode multiple proteins and are separated by an internal ribosome entry site (27). Intriguingly, many members of *Dicistroviridae* actually have a third, very short open reading frame that overlaps the second open reading frame (28). The function of the peptide encoded by this overlapping reading frame is not known, but clearly it is critical for virus function given the selective pressure that must be required to retain such a feature (29).

*Iflaviridae* are also positive-strand RNA viruses whose genomes contain many of the same proteins as the *Dicistroviridae*, including an RNA-dependent RNA polymerase. *Iflaviridae* are closely related to the *Flaviviridae*, which include several important human viruses, such as West Nile, Zika, dengue, and yellow fever viruses. There is just one open reading frame in these viruses; however, its translation generates one very large polyprotein that is then cleaved by a virus-encoded 3 C protease (30). While the functions of each protein are generally understood, how and why these viruses cause disease in bees are not well studied. Nonetheless, deformed wing virus (DWV; subtypes A and B) is perhaps the best-known honey bee virus among beekeepers. If acquired during development, infections can cause wing deformities that are easily recognizable when examining a honey bee colony (31); however, infected adults without deformed wings are common, as infections may also be acquired either during adulthood after wings are fully developed or during development at a low enough titer to avoid clinical symptoms.

There are many mechanisms, both horizontal and vertical (32), by which viruses can be transmitted between bees. Perhaps the most significant, at least as far as humans’ management of honey bees is concerned, is via *Varroa* parasitization. This is because the mite serves as not only a mechanical vector but also a potent biological vector in which DWV-B (and likely also DWV-A) can replicate (17, 33), thus amplifying virulence. *Varroa* mites can move between honey bees within a colony and, if they ingest a virus from one honey bee and then feed on another, they can transfer the virus. However, the mites can also travel between colonies via drifting or robbing workers, potentially spreading viruses to the new colony as well. While *Varroa* does not necessarily govern the transmission of all honey bee viruses, in theory any virus that can be carried by the mite and is infectious upon injection could be vectored. For example, black queen cell virus (BQCV) is not thought to be primarily transmitted by *Varroa* (34), yet in a large-scale observational study, BQCV titers do positively correlate with *Varroa* population (35). However, the effect varies depending on the year, perhaps in part because BQCV is also transmitted orally in a mechanism that is ostensibly dependent on the microsporidian parasite, *Vairimorpha apis* (36), which itself covaries by seasonal weather conditions (37). This example highlights the complexity of the honey bee pathogen and parasite web and how direct relationships between two players could go undetected if additional covariates are not accounted for.

### Bacterial Pathogens

Honey bees forage for nectar and pollen from a wide variety of plants, and they forage for water from ponds and puddles. Through these activities, they encounter multitudes of different bacteria species. However, there are really only two species of bacteria that are of any substantial detriment to honey bee health: *Paenibacillus larvae* and *Melissococcus plutonius*, the causative agents for American and European foulbrood diseases, respectively. As the common names suggest, both of these pathogens attack the larvae of honey bees, causing almost certain death if an infectious threshold is reached (38), with the resulting diseased larvae exuding a foul smell. In fact, the shift from pleasant aromas of resins, honey, and beeswax of a healthy hive to a distinctly unpleasant, rotten odor is often the first sign of trouble that an astute beekeeper detects. If a larva survives to adulthood, it will no longer be at risk of becoming diseased from either pathogen, but adult honey bees can carry the pathogens in their guts (though their health does not seem to be impacted) (39).

Both pathogens are highly infectious and of constant concern to beekeepers. Indeed, *P. larvae*, which was first described in 1906 (40), is the oldest known pathogen of *A. mellifera*, and *M. plutonius* was described shortly after in 1912 by the same scientist (Gershom Franklin White) (41). Larvae are exposed to these pathogens when they are fed by adult bees who harbor the bacteria, but they are only appreciably susceptible in the first few days of life (42). Resistance increases sharply thereafter, a pattern that coincides with a spike in phenoloxidase activity as the larva ages (see Layers of Immunity in Honey Bees) (43). Adult bees can also pass the bacteria from one to another via trophallaxis, or mouth-to-mouth feeding, and from colony to colony through drift, robbing, or contact with other bees during foraging. *P. larvae* is incredibly infectious, with just 10 spores being sufficient to cause disease (42), whereas *M. plutonius* is considered an opportunistic pathogen with variable virulence that depends on other factors, such as bacterial strain, nutritional stress within the colony, gut pH, as well as possibly rainfall and interactions with some fungicides (44–50).

As bacteria, *P. larvae* and *M. plutonius* can be controlled with antibiotics (tylosin and oxytetracycline) to some extent. However, in the case of *P. larvae*, which is spore forming and highly contagious, by the time an infection is detected, it is often too late for treatment. Some beekeepers use antibiotics prophylactically, but this is obviously an unsustainable practice, since antimicrobial resistance has emerged several times, especially in *P. larvae* (51). To help prevent prophylactic use, legislation in Canada and the United States requires veterinary prescriptions for antibiotics intended for colony treatment. Moreover, antibiotics are only effective at killing actively replicating bacteria; therefore, *P. larvae* spores, which can remain dormant in hive equipment for decades (52), are unaffected. While there are colony management techniques that can provide some measure of control, if infected colonies are identified, beekeepers may be required to euthanize the colony to comply with government regulations, which exist in the interest of protecting the broader population (48). *M. plutonius* does not form spores and is thus more easily remedied with antibiotics, but some jurisdictions still recommend euthanization in severe cases.

## Layers of Immunity in Honey Bees

### Innate Immunity

**FIGURE 1.**
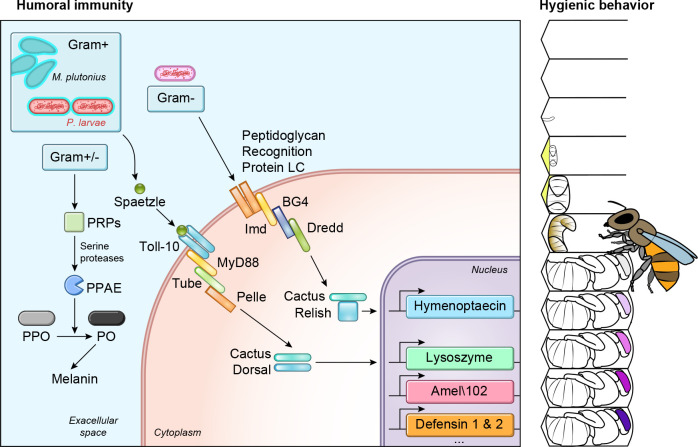
**Defense pathways against bacterial pathogens** As insects, honey bees have the canonical Toll and Imd pathways, which lead to expression of antimicrobial peptides, lysozyme, and phenoloxidase, all of which can kill bacteria. Some honey bees also exhibit hygienic behavior and related social immunity traits, where larvae or pupae that are diseased or dying are removed from the colony. PRP, peptidoglycan recognition protein; PPO, prophenoloxidase; PPAE, prophenoloxidase processing enzyme; PO, phenoloxidase.

Honey bees have a similar innate immune system to other arthropods and, indeed, most eukaryotes (19, 53), though not without divergent features (FIGURE 1). Like us, honey bees have physical barriers to infection and when those barriers are breached, infection risk is heightened. Puncturing the exoskeleton triggers a cascade that ultimately activates phenoloxidase. Among other immune functions, this enzyme catalyzes the key step in the synthesis of melanin, which is deposited around the site of injury or infection (54). Targeting of the melanization to the specific region impacted seems to be mediated by another protein, Amel\102, a homolog of P102Hv in *Lepidoptera* that facilitates amyloid fiber formation (55). Melanization works like blood clotting in mammals, in that it can function to seal a wound, but it also has other important effects. Bacteria, for example, become encapsulated in melanin, which impedes their movement and reproduction, and cytotoxic compounds produced during the reaction cascade help kill the invading bacteria (56, 57).

In parallel, bees can also produce several antimicrobial peptides in response to infection (FIGURE 1). As with other immune effectors, these peptides can be produced in several tissues, but the fat body seems to be the most important site of synthesis (58). Expression of the apidaecins (59) and defensins are controlled by the Toll pathway (Toll-like receptor in mammals), while abaecin and hymenoptaecin expression is downstream of the Imd pathway (similar to the NF-κB pathway in mammals, though with some Toll pathway cross talk) (19, 60). Interestingly, constitutive expression of these antimicrobial peptides is stimulated and maintained by the core gut microbiome (61). *A. cerana* expresses a notably greater number and diversity of antimicrobial peptides than *A. mellifera,* possibly due to their long evolutionary history with multiple pathogen-vectoring parasites (62). Phenoloxidase-induced melanization and antimicrobial peptides seem to be effective against virtually all bacteria that potentially threaten bees, but their expression is also stimulated by viral and fungal infections (19, 63). Evidence gleaned from other species suggests these effectors have the potential to help defend against nonbacterial pathogens (64), but this idea is not always supported (65); moreover, at the other end of the spectrum, some honey bee pathogens inhibit host immunity, resulting in a net decrease in immune effector expression (66).

Adult honey bees are rarely affected by bacterial pathogens, and *P. larvae* and *M. plutonius* have evolved mechanisms to bypass immune defenses by instead attacking larvae at a very young age. While a detailed mechanism is not yet understood for *M. plutonius*, we understand more about *P. larvae.* Honey bee larvae are most susceptible to *P. larvae* when they are very young, between 1 and 3 days after hatching. An examination of how the proteome of larvae changes with age revealed that phenoloxidase is not expressed at significant levels until day three, which was consistent with observations that only at day 3 could larvae mount a melanization cascade (43). Thus *P. larvae* seems to have evolved to attack honey bees during the one very small window when they cannot mount an effective response. It is likely that *M. plutonius* is a successful pathogen for the same reasons, and its success also creates a niche in which secondary bacterial invaders can thrive (67). Similarly, *Ascosphera apis*, a fungus, also appears to take advantage of the relatively defenseless, early stages of larval development.

### RNA Interference

**FIGURE 2.**
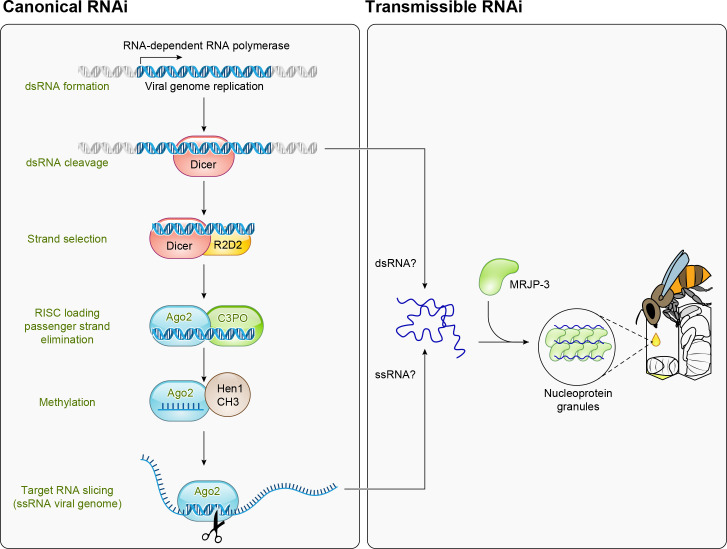
**Defense pathways against viruses** The RNA interference (RNAi) system is the main antiviral pathway in honey bees. It can be stimulated directly, by detection of double-stranded (ds)RNA, but RNA can also be passed from a worker to a larva, priming its RNAi pathway to be able to respond to the virus without having previously been exposed to it. MRJP, major royal jelly protein; RISC, RNA‐induced silencing complex.

Other immune defenses are crucial for bees’ defense against viruses, namely, the RNA interference (RNAi) pathway and heat-shock proteins (FIGURE 2). While RNAi is broadly considered to be a form of innate immunity, in honey bees it might be best considered as something in between innate and adaptive, owing to its systemic nature, long-term response, and the remarkable ability for bees to share dsRNA molecules between themselves and developing larvae (68, 69) (see *Transmissible Immunity*).

Experiments capitalizing on RNAi to silence specific genes can be fruitful for probing gene functions, with the first example in bees, to our knowledge, appearing in 1999 (70), and exciting methods are being established that exploit gut symbionts as novel RNAi delivery vectors (71–74). However, double-stranded (ds)RNA is also a general immune stimulator and induces expansive off-target effects on thousands of genes, even when a noninvasive introduction method is used (75, 76), which muddies the interpretation of RNAi experiments. CRISPR-based approaches, though not without their own challenges, seem more likely to lead to cleaner experiments with fewer confounding effects (77). Before these off-target effects were reported, one research group tested using dsRNA constructs targeting Israeli acute paralysis virus as veterinary antivirals for honey bees, with exciting results (78), but to the best of our knowledge, this technology has not yet received regulatory approval.

Despite RNAi being, by now, a well-characterized pathway, new protein participants are still being proposed. Some heat-shock proteins [e.g., Pl(2)el, HSC70-4, and HSP90] appear to have antiviral roles in honey bees, and workers clear infections faster if they are subsequently exposed to heat (79). These results are correlational but supported by mechanistic experiments in *D. melanogaster* (80). The processes underlying antiviral HSP activity in honey bees are unknown, but drawing on knowledge from mammals, HSPs might assist with activation or assembly of the RNAi machinery and participate in immune signaling (81), but much is still unevaluated. Regardless of the mechanism, the emerging role of temperature-dependent HSPs as antiviral immune effectors raises intriguing questions about interactions between honey bees and viruses in our changing climate.

### Social Immunity

When the honey bee genome was first sequenced, one of the surprising findings from comparative genomics analyses was the apparent contraction in the breadth of the canonical families of immune-related genes, relative to the other two insect species, which were sequenced at the time (3). Interestingly, taxonomically restricted honey bee immune proteins have since been identified, and presumably there are others yet undescribed, which may in part explain this ostensible restriction (82, 83). At the time, it was proposed to be a result of additional layers of protection afforded by the social structure of a colony, thus relieving selection for innate defenses alone. This idea has since been refuted (84), but the social immunity behaviors underlying the assertion are no less important or intriguing.

#### Hygienic behavior.

**FIGURE 3.**
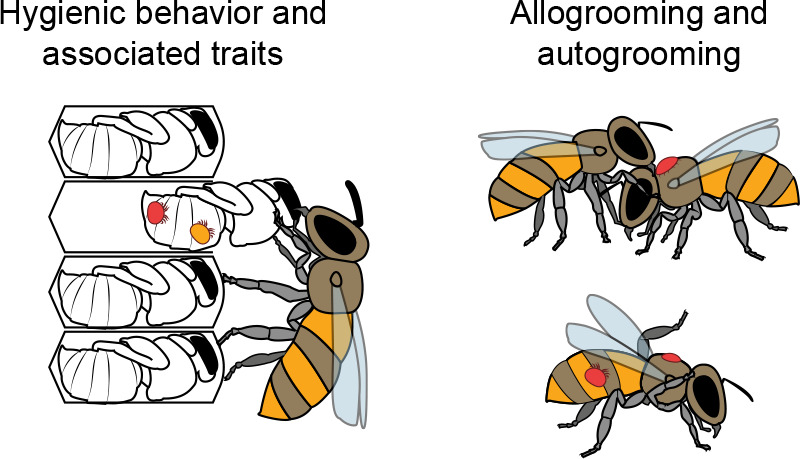
**Defense pathways against mites** Some honey bees exhibit social mechanisms of *Varroa* mite resistance. Grooming behavior and several behaviors related to hygienic behavior (but which target mite-infested cells, in particular) all function to keep mite populations under control, either by removing infested brood, disrupting the lifecycle of the mite, or removing mites from their bodies.

Aptly named, hygienic behavior refers to the removal of larvae or pupae that have been killed or damaged in some way (FIGURE 3). Hygienic honey bees seem to cue on specific scents given off by a dead brood, leading to terms such as “necromone,” meaning a pheromone given off by a bee during or after death, triggering its removal (85). Several volatile compounds have been suggested to be necromones, with little general agreement between studies (86–88). The lack of consensus indicates that not only are there multiple possible cues but also that there could be more than one odorant pathway that can trigger this behavior (11). Hygienic behavior does have a strong genetic basis, however, which has allowed researchers to successfully breed for this valuable trait. Similar behaviors (89–91) have also been described but we will not address them further here, as they may or may not be variants of the same behavior.

Beyond knowing that it is triggered by specific odors given off by dead or damaged brood and that the response is enhanced by octopamine (92), we do not know much more about the conserved mechanistic basis of this behavior. Genetic and genomic attempts to identify causative genes have yielded little consensus, except to determine that the trait is polygenic (11). This is despite honey bees having a very high rate of genetic recombination [among the highest of all animals (93, 94)], which should actually increase the likelihood of identifying causal mutations. We have previously speculated that the lack of consensus between studies on hygienic behavior and other social immunity traits may be a result of the traits not only being highly polygenic but also having variable genetic underpinnings (that is, multiple genetic pathways could result in the same behavior). For example, multiple studies have linked odorant binding proteins to hygienic behavior, but do not agree on exactly which ones are important (11). This might be because different odorant binding proteins enable detection of different odorants, and in theory detection of any of a multitude of odorants associated with death or disease could be sufficient to trigger the behavior. As modern, high-resolution sequencing efforts are ever more accessible, we hope that, combined with the asset of high-frequency genetic recombination, these questions will soon become resolved.

#### Grooming.

Grooming refers to a behavior where some honey bees are especially proficient at removing *Varroa* mites from their own bodies or those of their nestmates, similar to the tick-grooming behavior described in some primates (95). Typically, grooming bees will also damage the mites they remove, although we do not yet understand whether this is done intentionally or incidentally. Either way, colonies with higher levels of grooming tend to have lower levels of *Varroa* mites (96). Grooming appears to be heritable, which has led researchers to try to breed for this trait, similar to hygienic behavior (97). While genes linked to behaviors are frequently pleiotropic and genetic relationships between social immunity traits are possible, in the absence of causal mechanisms, there is not yet concrete evidence supporting pleiotropy here. What causes some bees to groom better than others is still largely unconfirmed, although proficient groomers have larger mandibles than poor groomers (98).

#### Self-medication.

Emerging evidence suggests that bees could be among the many animals in which zoopharmacognosy, or self-medication, has been observed (99). In addition to foraging for pollen, nectar, and water, *A. mellifera* workers forage for resin from trees and buds. When they return to their colony, they mix this resin with wax and saliva to create propolis, which they then use to fill cracks in the structure of their hive. However, new evidence suggests that the bees could be self-medicating. When Simone-Finstrom and Spivak (100) challenged bees with *A. apis*, they observed that the bees increased resin foraging rates, and the presence of a propolis envelope has been linked to *P. larvae* and *A. apis* resistance (101, 102). Compounds in poplar (*Populus*) resins in particular appear to be among the active molecules against both pathogens (103). A similar behavior has been observed in some bumble bee species where parasitized bees displayed a preference for food (either naturally foraged or human supplied) containing alkaloids (e.g., nicotine) or glycosides, both of which can reduce parasite loads or slow parasite development (104–106). As with so many behaviors in bees, the exact mechanism(s) underlying these responses to pathogens are unknown.

### Transmissible Immunity

As invertebrates, bees have neither an adaptive immune system nor any of the memory cells we associate with adaptive immunity in mammals. Nonetheless, like many other insects, honey bees can pass information about some previous bacterial (107) and possibly viral (108) encounters vertically from parent to offspring through transgenerational immune priming. Queens exposed to inactivated *P. larvae* convey greater tolerance of that pathogen to their offspring by passing on fragments of the pathogen to the developing zygote (107, 109), where it likely stimulates immune pathways via proteins that recognize pathogen-associated molecular patterns. At a minimum, this process involves the protein vitellogenin chaperoning those fragments during oviposition (109), with the fragments stimulating hemocyte differentiation in larvae (107).

In addition to vertical transmission of bacterial immune elicitors, other immunological agents can be transferred diagonally (between adult workers and the developing kin they tend, as opposed to strictly horizontally between adult workers). The expression of defensin-1, but not defensin-2, in the glandular organs that produce and secrete the jellies fed to queens and developing larvae (110) as well as the jellies themselves (111) suggests the effector could be shared between individuals or prevent contamination of food when larvae are most vulnerable. Similar functions are described for other secreted antimicrobial peptides (jelleines) (112).

More concrete and exciting evidence exists for the transmissibility of dsRNA, however, which then triggers an RNAi response in the recipient bee. When dsRNA is ingested by adult workers, it spreads systemically within the bee and eventually is secreted and delivered to larvae via their jelly diet (69). The dsRNA is subsequently ingested by the larva, where, remarkably, it remains biologically active (69). This transfer from worker to larva is mediated by major royal jelly protein-3 (113), but, as honey bees do not possess an RNA-dependent RNA polymerase gene, the mechanism of systemic dsRNA proliferation remains unknown.

## Xenobiotic Exposure in Honey Bees

*A. mellifera* foragers can fly as far as 5 km from their hive to find food and they may visit hundreds of blossoms during that flight, although much longer flights have been documented (114). Once the transition from nursing to foraging has been made, nearly every forager will do this for the remainder of its life. With ten thousand or more foragers in a colony, honey bees sample nearly every food source in their neighborhood and are exposed to a wide range of xenobiotic chemicals, both naturally occurring and synthetic (115). Some of these exposures can be lethal, either to an individual bee or to the whole colony; although rare, these events tend to make news headlines and so we hear about them disproportionately. Much more common and insidious is sublethal exposure, which can still have devastating impacts on bee health that we are only just coming to understand (116).

In North America and Europe, all chemicals that can legally be used in agriculture or other outdoor applications must now be tested on honey bees, as well as on many other organisms upon which they could have a negative impact. These studies tend to only evaluate lethality, with virtually no examination of sublethal effects. In addition, these studies focus on honey bee workers, ignoring possible effects on the queen or drones (reproductive individuals). We will discuss here what is known about the impact of these on honey bees, but it is vital to note that there are many other important species in the ecosystem, with varying sensitivities, that are never considered when regulatory approval of a new compound is being contemplated.

### Agrochemicals

In 2009, the latest year we could identify collated data, there were approximately 800 different active compounds registered for use in crop protection around the world (117). This does not include other compounds farmers might apply, such as fertilizers, growth stimulants, adjuvants, etc. It also does not include compounds used in veterinary practice or other industrial chemicals that are not used in agriculture but that bees might still be exposed to through their sampling of the environment. Thus, beyond the naturally occurring toxins encountered on plants, bees can encounter a wide range of anthropogenic toxins as well. Here, we will focus on broad groups of compounds that are the active ingredients in pesticides that honey bees might encounter on crops.

#### Fungicides.

Antifungals are, unfortunately, commonly applied to crops during bloom in the hopes of killing any fungi that might infect and ruin the fruit that is about to develop. Bees will visit the blooming flowers at this time, too, so exposure is almost certain. In fact, fungicides are often the most prevalent pesticide found inside the hive (118, 119). The proteins targeted by fungicides do tend to be specific to fungi, but off-target effects have been documented in honey bees (120). In addition, honey bees have a microbiome that includes commensal fungi (121), which can be impacted by fungicides, potentially imparting an indirect effect on their health via altered nutrient uptake and immunity.

#### Herbicides.

It may seem counterintuitive that bees should be exposed to herbicides since, if they are effective, they should kill the plants before bees visit them. However, sometimes plants can still produce flowers as a last-ditch effort to pass on their gametes after herbicide exposure. In other instances, farmers or gardeners could attempt to target certain plants by manual spraying, with nearby blossoms receiving lower but still significant levels of the compound. All of these compounds can end up in groundwater, leading to another route of exposure to bees. Another significant source of exposure comes from Roundup Ready crops produced by Monsanto. These crops have had a mutant version of 5-enolpyruvylshikimate-3-phosphate synthase inserted into their genome, which allows them to survive in the presence of glyphosate, the active ingredient in the herbicide Roundup. The advantage of these crops for farmers is that they can apply Roundup to control weeds without killing the crop. The disadvantage is obvious: this can be problematic to the rest of the ecosystem, likely including bees [though notably, the nature of negative effects has been mixed and hotly debated (122, 123)].

#### Insecticides.

The impact of fungicides and herbicides on bees is unintentional, largely the result of off-target effects on bee proteins or commensal microbiota. Insecticides, on the other hand, are specifically designed to kill insects. To be registered for use, insecticides are supposed to impact the target species with minimal effects on beneficial, nontarget species, including bees. This recognition that we do not want to kill all insects when we apply an insecticide has only developed relatively recently: older classes of insecticides tended to be indiscriminate. However, in order for an insecticide to be effective, the protein(s) that it targets has to be essential to insect life, which typically means that they are also found in bees. The most widely used class of insecticides in recent decades has been the neonicotinoids, which largely replaced all previous classes of insecticides used in agriculture.

Neonicotinoids are a group of neuroactive nicotine derivatives that target the nicotinic acetylcholine receptors. These systemic pesticides are absorbed into a plant and then spread throughout the whole organism, including into nectar and pollen. When an insect pest feeds on the plant, it ingests the insecticide and is killed. Bees also feed on plants, albeit in a way that is beneficial, but they still end up being exposed to the systemic insecticides. The doses bees receive should not normally be lethal; however, numerous sublethal effects have been observed (124), and there is still a large knowledge gap in this area (116). The sublethal effects are not just limited to neurological effects: neonicotinoids also impair the immune system of honey bees, with reports linking exposure to suppressed antiviral activity, reduced hemocyte density, deficiencies in melanization, and lowered antimicrobial activity of hemolymph in *A. mellifera* (125–128). In a worrying demonstration, one neonicotinoid, clothianidin, has even been shown to reduce the bees’ ability to perform hygienic behavior (an important trait that helps colonies resist some brood diseases; see *Social Immunity*) (129). In light of these findings, it is unsurprising that numerous interactions between pesticide exposure and pathogen infections have been reported, which are reviewed elsewhere (130, 131).

#### Pesticide combinations.

When a new pesticide is tested for market, the compound is tested in isolation. However, bees are commonly exposed to multiple pesticides at once, as well as other sources of stress that could interact. The routes of exposure are not precisely known, but bee-collected pollen tends to have a variety of pesticides, more than in nectar or bees themselves (118, 119, 129, 132). Additionally, pesticides are rarely sold with a single active compound; for example, neonicotinoid seed dressing is patented with at least three different fungicides (133). This is critical since fungicides can synergize with neonicotinoids through inhibition or competition for detoxification proteins (134). However, the addition of a herbicide to the neonicotinoids does not appear to change the toxicity (129). Unfortunately, combinations of pesticides are not regularly tested, and it is not part of regulatory assessments.

### Bee Medications

Beekeepers try to protect their livestock from pesticides applied by farmers, but they also use other pesticides themselves to control bee pathogens. The primary medications beekeepers use are miticides, fungicides, and antibiotics. Any compound registered for use in bee hives has been tested for efficacy, and the recommended dose is adjusted to minimize the impacts on bees, but minimal impact does not mean zero impact. Coumaphos causes honey bee queens to die earlier and reduces the likelihood of acceptance into a colony (135–137), while *tau-*fluvalinate slows down queen development (136). These two synthetic acaricides used to control *Varroa* mites are now much less effective as widespread resistance emerged (118), and resistance has emerged for a third acaricide, amitraz, in some locations (138). There may be a trade-off for the mite in detoxifying at least *tau*-fluvalinate, however, as it can sometimes still be effective after not being used for several seasons, suggesting that the mites lose resistance. Other, more “natural” compounds are also used to control *Varroa*, such as thymol, formic acid, and oxalic acid. However, these are not necessarily any healthier for bees than the more synthetic compounds. Thymol seems to induce worker honey bees to remove brood (139, 140) and to increase queen mortality (141), while the organic acids can also impact worker (142) or queen longevity (143) and brood viability (144). Thus, while all of the compounds mentioned here tend to have a favorable cost-to-benefit ratio in terms of treating bee diseases, none are completely benign to honey bees.

## Honey Bee Detoxification Mechanisms

The honey bee genome encodes for five superfamilies of detoxification systems: cytochrome P450 monooxygenases, carboxylesterases, glutathione *S*-transferases, UDP-glycosyl transferases, and ATP-binding cassette transporters (145). These systems are common to virtually all animals, and the encoded proteins are responsible for breaking down a wide variety of exogenous and endogenous chemicals. Interestingly, most of the detoxification genes are highly expressed in the larval stage (145), which is puzzling because in pesticide residues the larval food produced by workers is largely detoxified, with a >500-fold lower hazard quotient than the pollen consumed by the workers (146). However, some pollen is mixed with the larval diet at later instars, which may be sufficient to necessitate high detoxification enzyme expression. Expression then decreases during the pupal stage, when bees are not ingesting any new food and are protected from wax residues within a silk cocoon, making them unlikely to encounter xenobiotics. When they emerge as adults, the expression of most detoxification enzymes increases again to prepare them to face the onslaught of foreign chemicals they will encounter when foraging.

By far the best-studied detoxification pathways in honey bees are those involving cytochrome P450s. There are four major clades of P450s based on amino-acid similarity (CYP2, CYP3, CYP4, and mitochondrial P450s) (147). In other insects, CYP2 and mitochondrial P450s are largely involved in biosynthesis of molting hormones, while CYP4 genes are involved in xenobiotic processing and juvenile hormone synthesis, and CYP3 genes metabolize xenobiotics (148). In honey bees, the CYP3 clade has been confirmed to detoxify xenobiotics (149), with the CYP9Q subfamily seeming to be key for detoxifying neonicotinoids (145, 150), organophosphates, and pyrethroids (151), among which are the insecticides understood to be most damaging at this time. The phylogenomic diversity of this subfamily seems to underly some of the varying sensitivity to neonicotinoids seen in various bee species (152, 153).

Carboxylesterases have been implicated in detoxification of miticides (154) but remain understudied. Of the 24 carboxylesterase-coding genes, 8 are associated with detoxification (147), but only one has been directly implicated in detoxification in honey bees (155, 156).

Glutathione-*S*-transferases are also divided into classes based on amino-acid similarity (157). Honey bees have only one delta class gene, which has been implicated in detoxification (156, 158). The sigma and omega classes were also upregulated in honey bees exposed to a variety of toxins (159, 160), although this might have been a secondary response to oxidative damage caused by these toxins (159–162). The other classes, theta and zeta, probably play roles in specific metabolic processes, such as amino acid catalysis, among other functions (147).

ATP-binding cassette (ABC) transporters are thought to be important in insect detoxification and can be divided into eight subfamilies (ABCA to ABCH) (163). ABC transporters have been studied extensively in the context of multidrug-resistant (MDR) human pathogens (164); however, they are wildly neglected in bees, with only one study showing evidence of the importance of MDR transporters in miticide and neonicotinoid detoxification (165) and another showing that two MDR-associated genes were upregulated in bees fed with *p*-coumaric acid (155).

The microbiome community may convey resistance to or an ability to absorb/detoxify potentially harmful xenobiotics. In honey bees, acquiring the intestinal microbiome stimulates the expression of cytochrome P450s in the epithelium of the gut (166), which presumably then enables the bees to break down exogenous toxins. While there is not yet any evidence of direct detoxification by the microflora themselves, the microbiome does appear to play an important role in protecting honey bees from xenobiotics by promoting host enzyme expression.

## Future Outlook

This is not a comprehensive review of all honey bee pathogens; there are many and their biology has been covered elsewhere in more detail (19, 20, 48, 167–170). Likewise, there are thousands of xenobiotics that honey bees encounter and an exponentially larger number of potential interactions between xenobiotics, pathogens, and parasites (reviewed in Refs. 130, 131). We cannot discuss all of them, and, indeed, we do not even know how most affect host physiology. Rather, we have attempted to present some of the pathogens and agrochemicals that have the most significant impacts on honey bee health and where we also know something about both the molecular processes underlying the health impacts and how honey bees respond to them.

### Why Do We Not Know More?

A common thread running through this review is that we simply do not have a reasonable mechanistic understanding of the molecular basis for pathogenesis or defense in most of the topics described. This relative lack of information has meant that the development of the tools (e.g., antibodies, molecular biology techniques, genetic systems, and cell lines) that have been so critical in building our understanding of other biological systems has lagged behind for bees. The knock-on effect of this is that the level of mechanistic detail that we expect in contemporary human or model organism research is simply not yet feasible in honey bees. However, beyond research tool availability, *A. mellifera* is a more challenging organism to study than, e.g., *D. melanogaster*, mainly for the reasons discussed in the following sections.

#### A need to be free.

*A. mellifera* cannot be easily maintained in a controlled laboratory environment or even in a greenhouse. They need to fly, not only to forage for food but also to mate and defecate. In addition, they depend on the sun to govern their circadian rhythms, perhaps more than most other animals. In completely enclosed spaces with only artificial light, or even natural light within greenhouses, they are drawn to that light and tend not to return to their colonies. Outdoor flight cages have been used to some limited success, but honey bees still cannot thrive in these. From the perspective of trying to control variables in an experiment, however, having to keep animals outdoors, unrestricted, is obviously suboptimal. Not only can we not control their environment, but we cannot even control their food sources or their exposure to pathogens, so the risk of extraneous variables is high.

#### Mating and polyandry.

When a honey bee queen emerges as an adult, she goes on several mating flights where she will, optimally, mate with dozens of drones (171). This mating occurs during flight, tens of meters in the air, and the sperm she receives from her mates is stored in her spermatheca to be used for fertilizing eggs for the rest of her life (171). There are means by which to instrumentally inseminate queens using semen collected from drones, but queens fertilized in this way are less robust than naturally mated queens (172). Nonetheless, instrumental insemination is a critical tool for researchers to have some control over genetics, and it is used in some (though few) commercial beekeeping operations to maintain and propagate certain highly desirable lineages of bees. In the classical Mendelian approach that scientists use for genetics, however, we would normally want one female to mate with one male so that we know both the mother and the father of any offspring. The genetic diversity within a colony that results from having dozens of drones mate with one queen is critical to honey bee biology but is obviously problematic for classical genetic studies. With instrumental insemination, it is possible to create a viable queen using semen from just one drone but our instinct, as scientists, to reduce noise in the genetics by creating inbred lines conflicts with bees’ sex determination system.

#### Inbreeding.

As members of the Hymenoptera order, bees have a haplodiploid sex determination system (also known as arrhenotokous parthenogenesis). Canonically, this means that unfertilized (haploid) eggs develop into males, whereas fertilized (diploid) eggs turn into females. This is not strictly true, however: more critical is the zygosity of the complementary sex determiner (*csd*) gene (173). If a bee egg has only one copy of *csd*, it will develop into a male, but if has two identical copies of *csd*, it would also turn into a male. However, diploid males are abnormal and quickly cannibalized by workers upon hatching (174). Thus only larvae that are heterozygous or hemizygous at the *csd* locus reach adulthood. This intriguing mechanism, which was recently shown to depend on differential complexation of Csd protein variants (175), ensures genetic diversity in a population, but it also has enormous implications for honey bee research: the direct effect is that creating inbred lines of honey bees, which would be extremely useful for genetic research, is impossible.

### How Do We Solve the Knowledge Gaps?

The answer to how to fill knowledge gaps is always “more research.” However, we as a society, and particularly our funding agencies, need to appreciate the importance of fundamental research for building the knowledge base on which all future applied research depends. Only by supporting research into the intrinsic biology of all species of bees can we hope to build the understanding that we need to help these species survive disease and flourish in an anthropogenic world.

One advance that would help enormously would be the development of at least one resilient, easy-to-grow, immortalized cell line from honey bees or, indeed, any other species of bee. Our ability to grow cells (whether primary or immortalized) from other organisms ex vivo has been invaluable for advancing knowledge of molecular mechanisms. We have cell lines from many plants, fungi, and animals, yet despite considerable effort by many talented people, no one has yet created a robust, immortalized cell line from any bee species. Primary cells from some tissues can be maintained in culture for short periods of time (176, 177), but the opportunities with these are limited. The most successful attempt at an immortalized cell line (178) was promising, with several subsequent publications (179–182), but still resulted in something that has been difficult for others to propagate and is seldom utilized beyond the research group that developed it. This lack of an in vitro model has severely limited our insights into bee molecular and cellular biology.

As with so many areas of biology, CRISPR holds much promise for elucidating more molecular mechanisms in bees. Because of the complex genetics, inability to inbreed, lack of cell lines, and difficulty maintaining colonies in containment facilities, manipulating even one gene is still an enormous challenge and few groups have been successful (183). CRISPR itself is still challenging in honey bees with only a handful of studies yet capitalizing on the technology (reviewed in Ref. 183). However, as more researchers try it, we expect that the methods will become more robust and more accessible to the broader community.

### What Are the Most Important Gaps?

The large majority of molecular knowledge we have in bees comes from comparative genomic analyses where knowledge from *D. melanogaster* and even more distantly related species is ported to bees, with the assumption that molecular pathways probably function in the same way. This is understandable and necessary: if we did not do this and worked on every organism in a vacuum, without transferring knowledge based on evolutionary relatedness, science would be decades behind where it is now. This is especially true with bees, where the challenges in testing molecular mechanisms would mean that most of what has been discussed above would likely not yet have been discovered. However, comparative genomics does lead to a false sense of knowledge. Certainly, most of the fundamental “housekeeping” processes are likely to function similarly, but bees diverged from Diptera (flies) hundreds of millions of years ago and many gene functions have changed considerably over that time. Comparative genomics has not helped propose functions for the ∼3,000 “uncharacterized” genes and ∼4,000 genes with no gene ontology term associations that still exist in the *A. mellifera*, the best-studied bee, let alone the thousands of other species of bees. Moreover, the vast majority of genes that do have putative functional assignments have never been confirmed experimentally.

Bees have some fascinating and truly puzzling physiology and behaviors. While the challenges in understanding the mechanisms behind these are significant, advances made in other organisms and technological improvements are helping to shed light on this vital group of insects. However, unless we do the work to assign functions to genes through fundamental experiments, the plethora of physiological and systems biology data that already exist will continue to go underutilized. The contemporary challenge for advancing our knowledge of honey bee physiology is thus no longer generating the data, it is how to interpret them.

## Footnotes

^1^
We attempt to use “honey bees” whenever we mean at least one of the two species for which this is a common name (*A. mellifera* and *A. cerana*). We use the more generic “bees” when we are referring to some concept that is or could be common to many, if not all, bee species.

^2^
There are four species in the genus *Varroa*. By far the most economically important is *V. destructor*. The common name used for *V. destructor* is simply “*Varroa*” so we use the term in that way here, too.
